# Supplementary material for: The Gut Microbiota Profile in Children with Prader–Willi Syndrome
Source: Genes (Basel). 2020 Aug 7;11(8):904. doi: 10.3390/genes11080904 (PMC7463799; doi:10.3390/genes11080904)
Supplement: Supplementary file 1 [file genes-11-00904-s001.pdf]

SUPPLEMENTARY FIGURES AND TABLES

Figure S1: Importance of predictors. Importance of the identified taxa in models for (A) differentially abundant bacterial genera: PWS vs. CON, (B) differentially abundant bacterial genera: OWOB CON vs. NW CON, (C) differentially abundant bacterial genera: OWOB PWS vs. NW PWS, (D) differentially abundant fungal genera: PWS vs. CON, (E) differentially abundant fungal genera: OWOB CON vs. NW CON, (F) differentially abundant fungal genera: OWOB PWS vs. NW PWS, (G) predicting PWS using selected bacterial and fungal genera markers, (H) predicting OWOB in CON subjects using selected bacterial and fungal genera markers, (I) predicting OWOB in PWS subjects using bacterial and fungal genera markers. Predictors with grey labels in A-F were those with importance between the 80% and 90% quantile in each model and were not included in the final models.

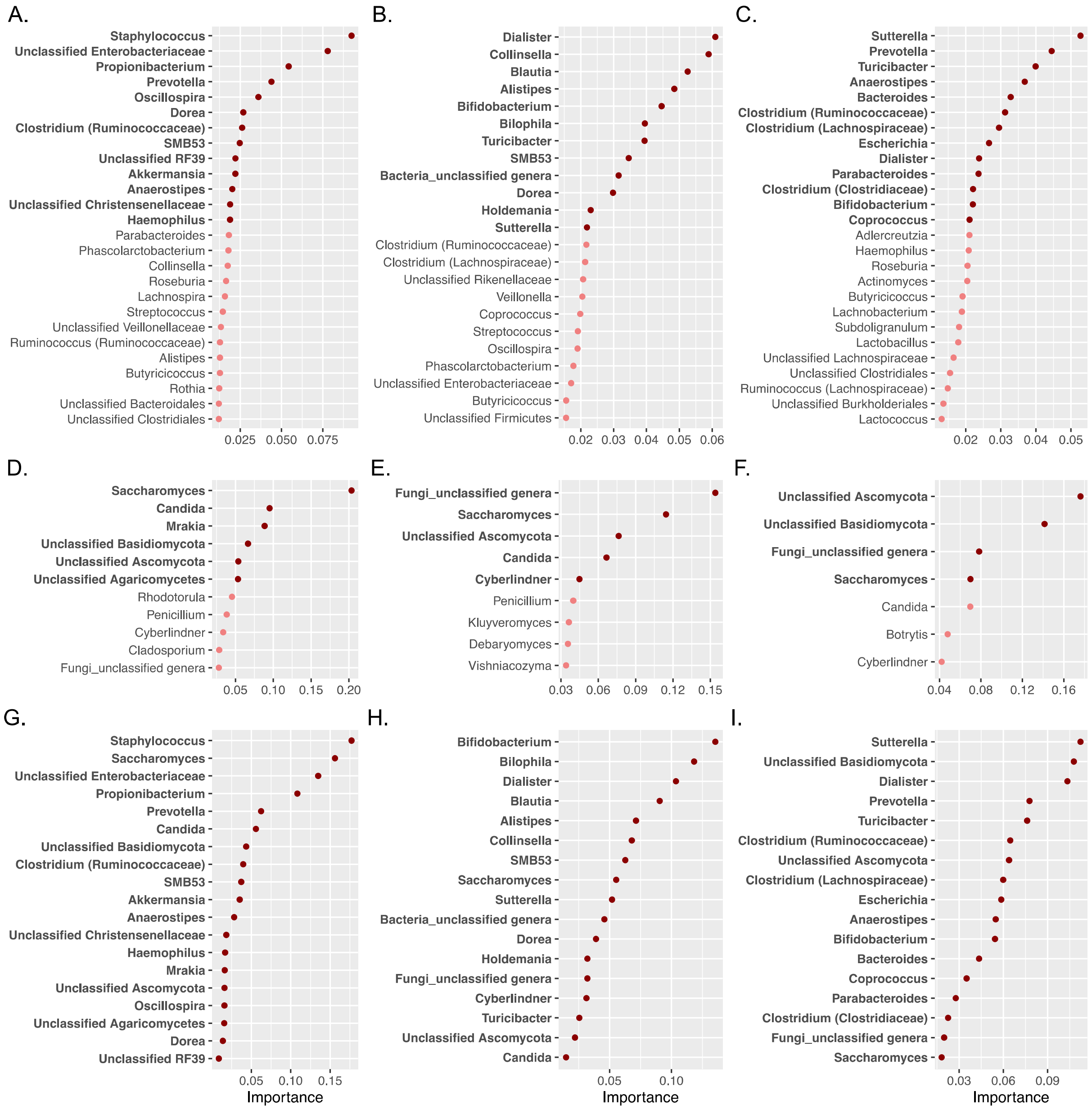

Figure S2: Performance of the model for differentiating PWS from CON using bacterial genera markers.

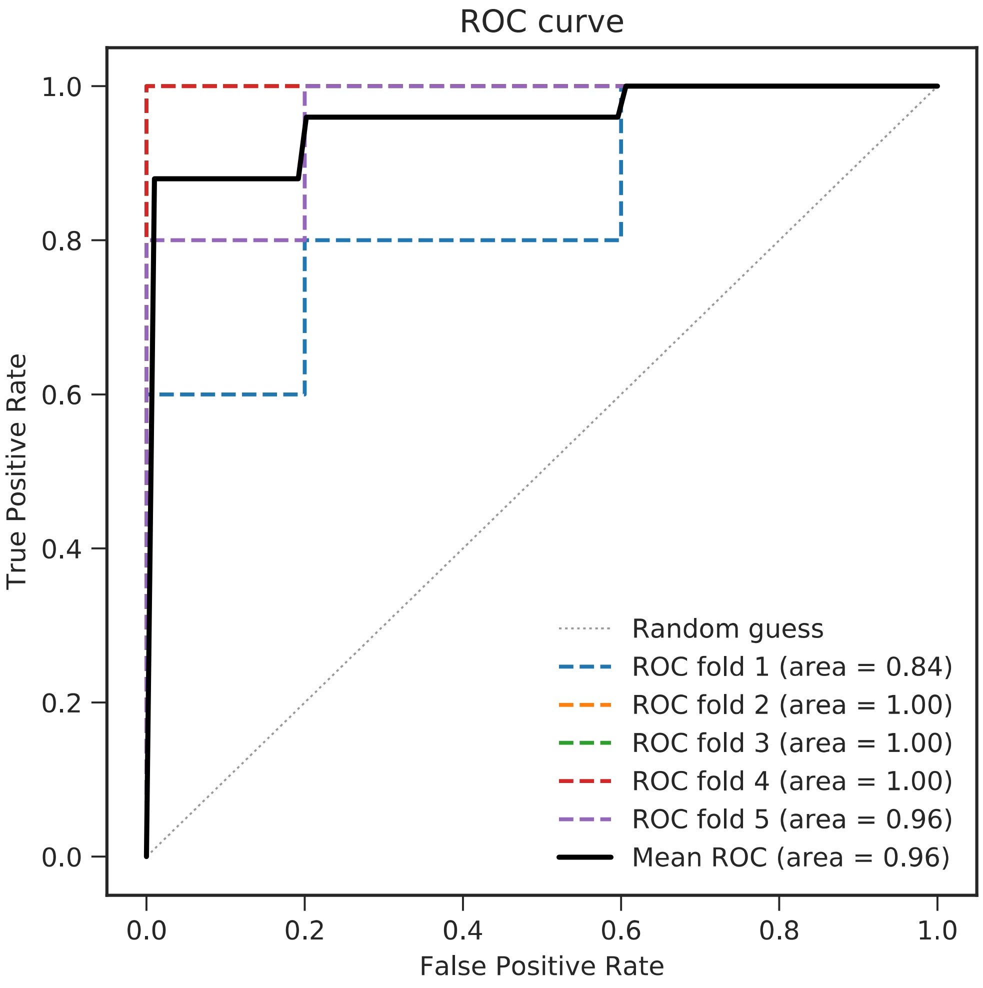

Figure S3: Performance of the model for differentiating OWOB CON from NW CON using bacterial genera markers.

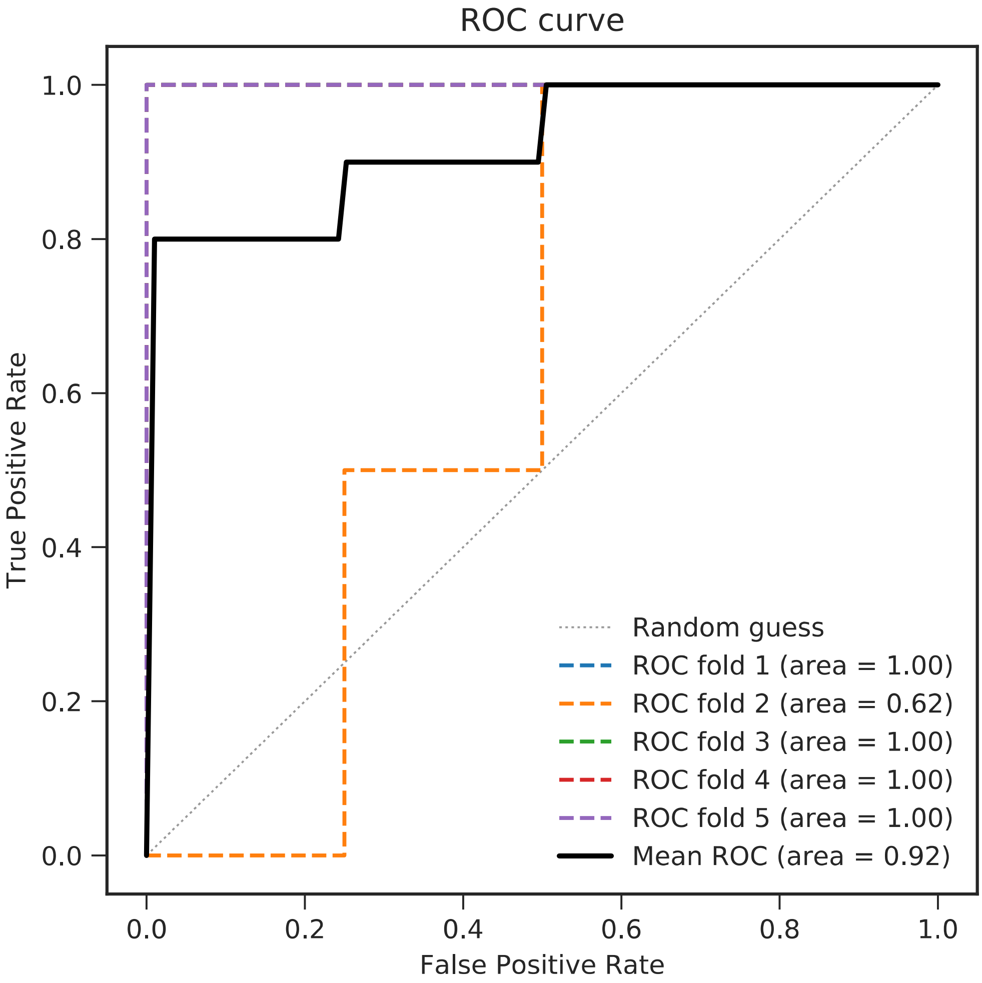

Figure S4: Performance of the model for differentiating OWOB PWS from NW PWS using bacterial genera markers.

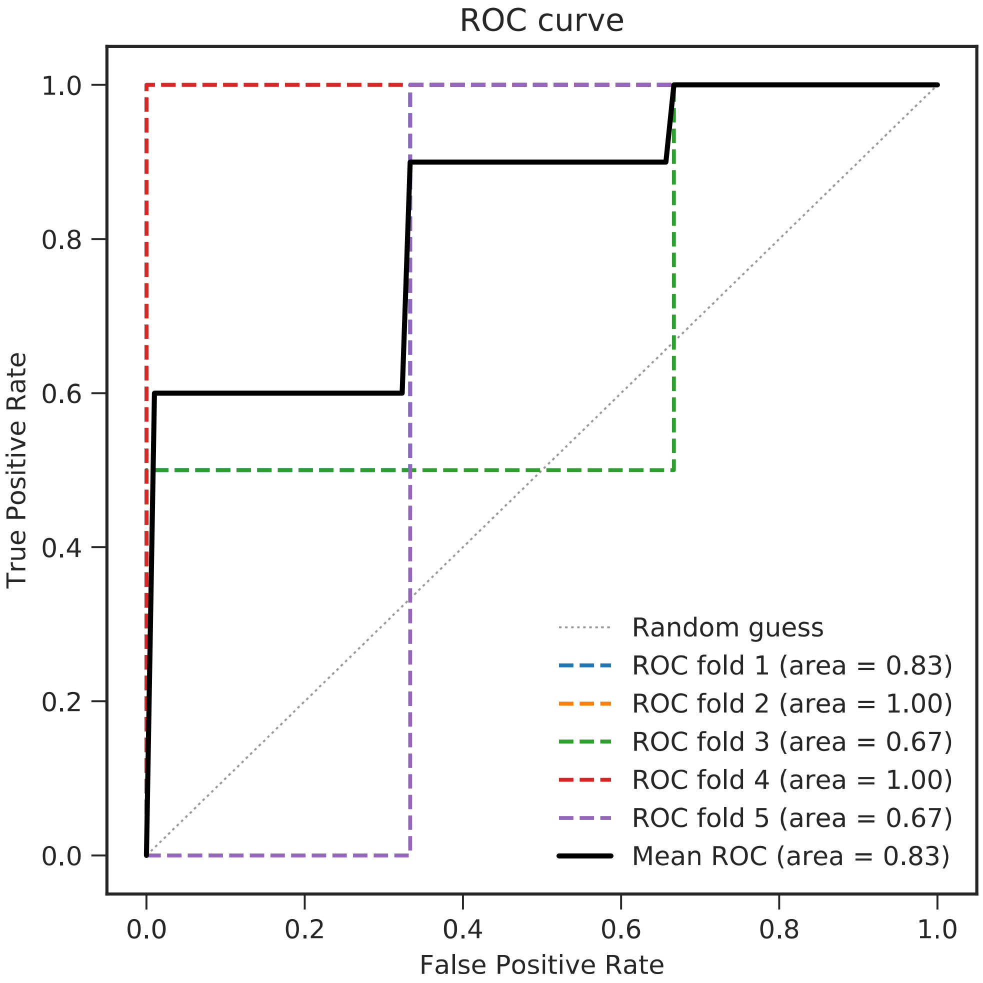

Figure S5: Performance of the model for differentiating PWS from CON using the identified fungal genera markers.

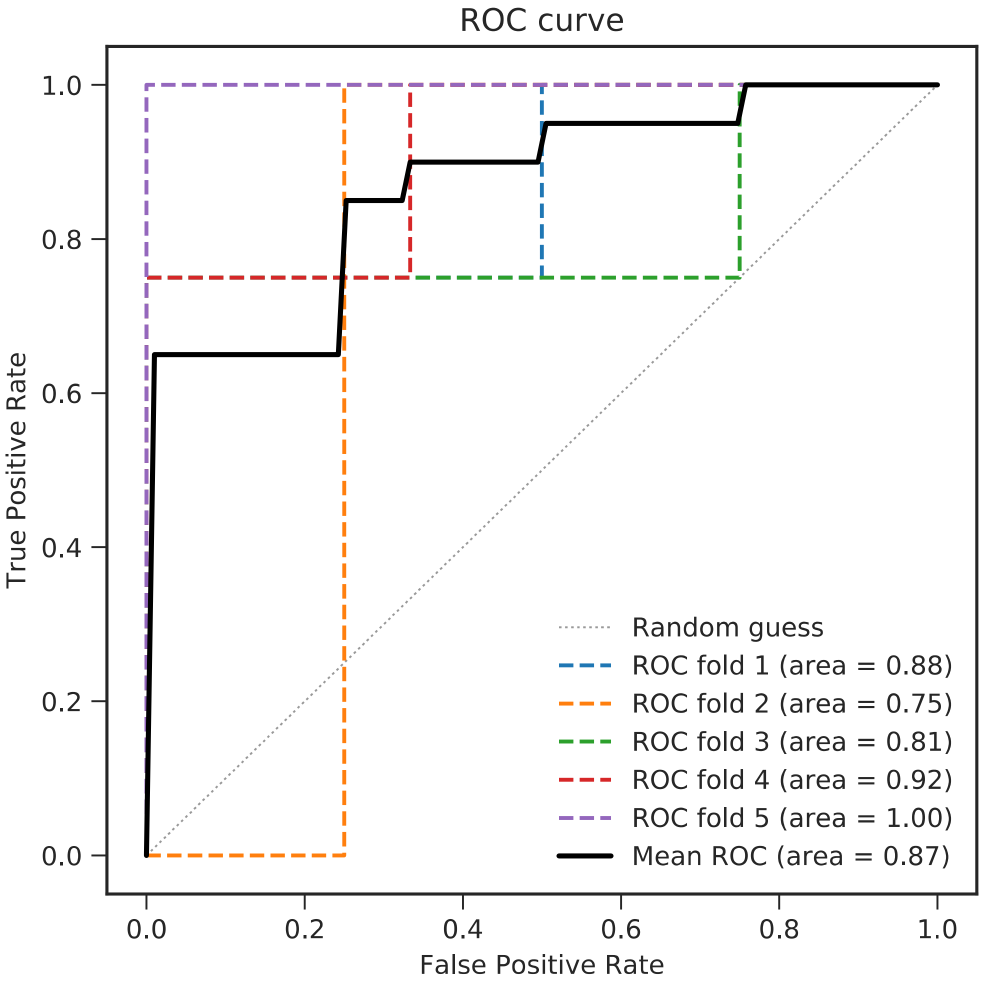

Figure S6: Performance of the model for differentiating OWOB CON from NW CON using the identified fungal species markers.

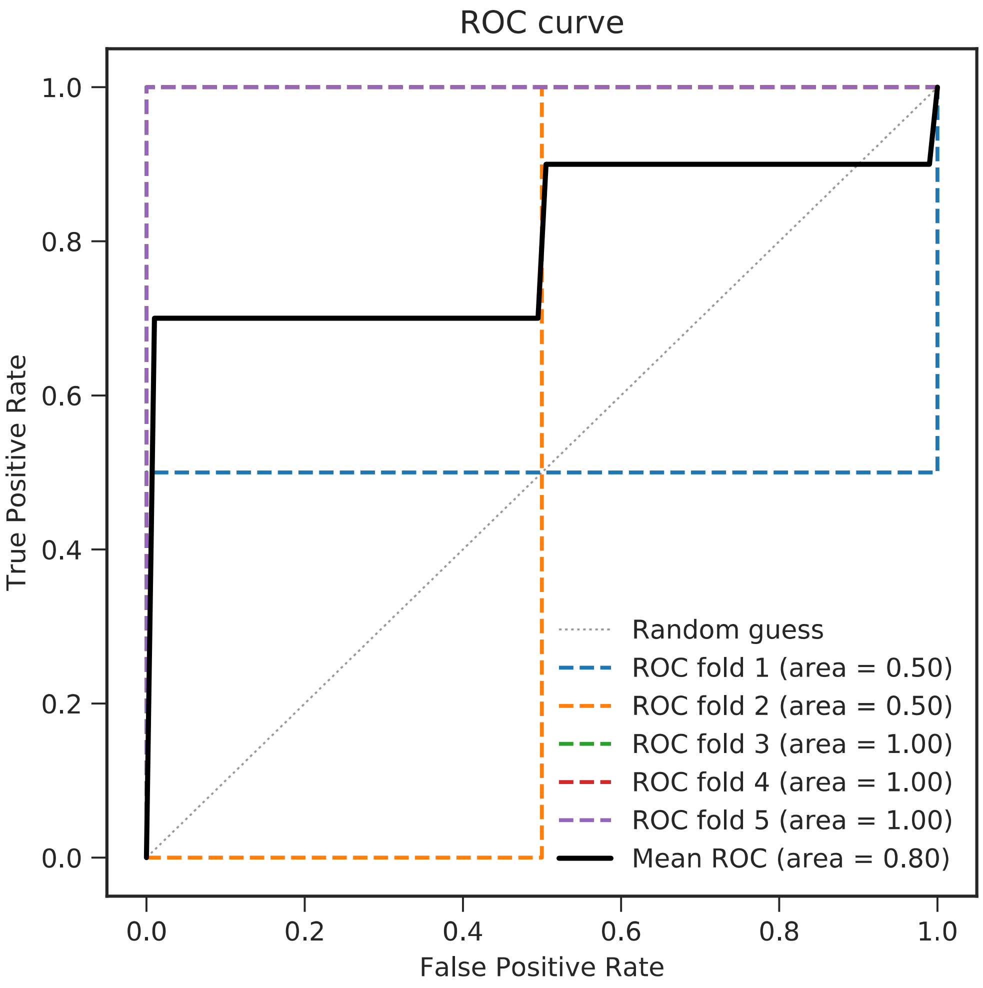

Figure S7: Performance of the model for differentiating OWOB PWS from NW PWS using the identified fungal genera.

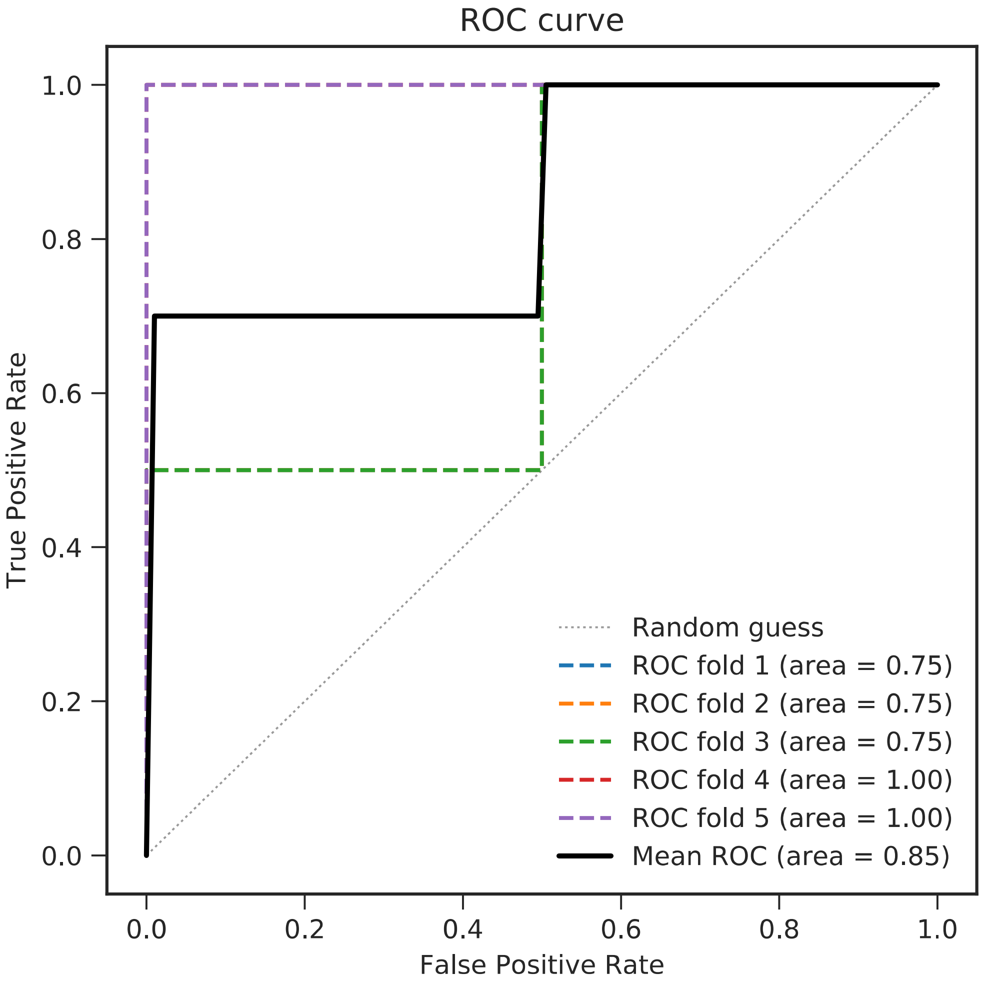

Figure S8: Performance of the model for differentiating PWS from CON using the identified bacterial and fungal genera.

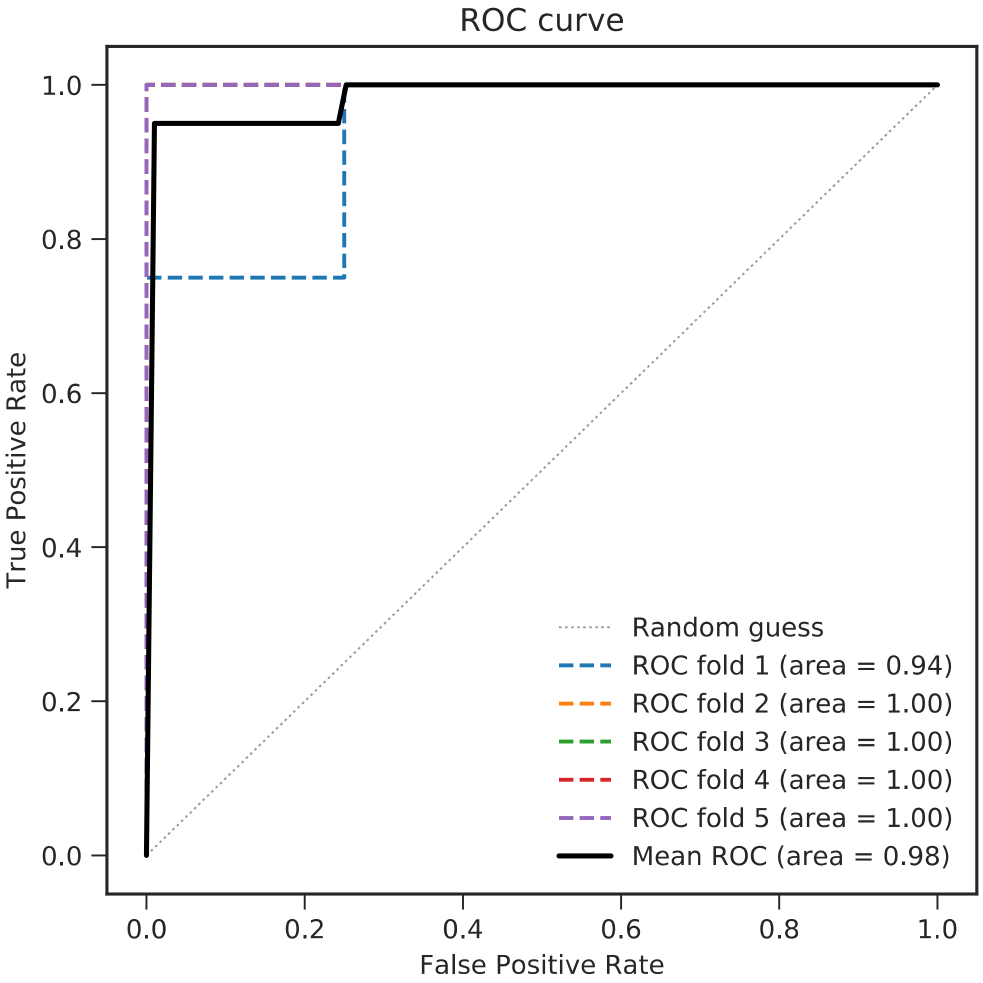

Figure S9: Performance of the model for differentiating OWOB CON from NW CON using the identified bacterial and fungal genera.

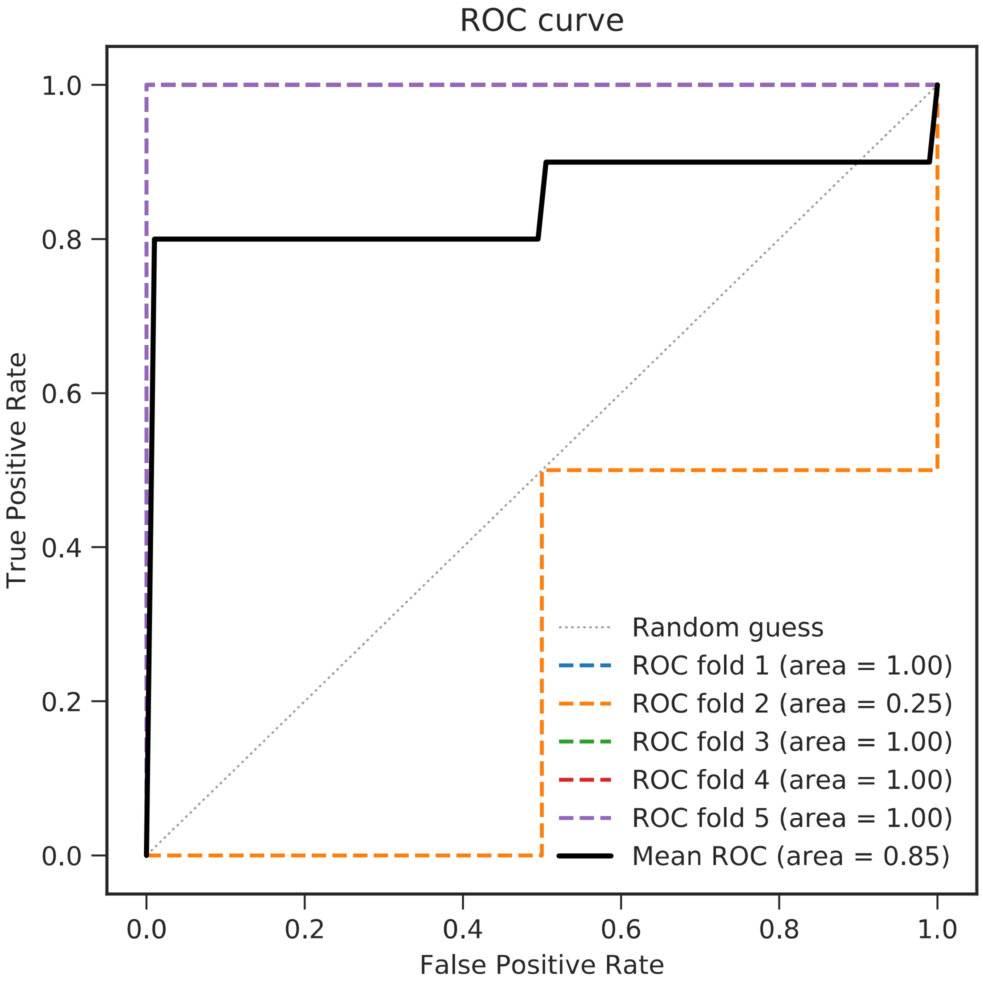

nera.

Figure S10: Performance of the model for differentiating OWOB PWS from NW PWS using the identified bacterial and fungal genera.

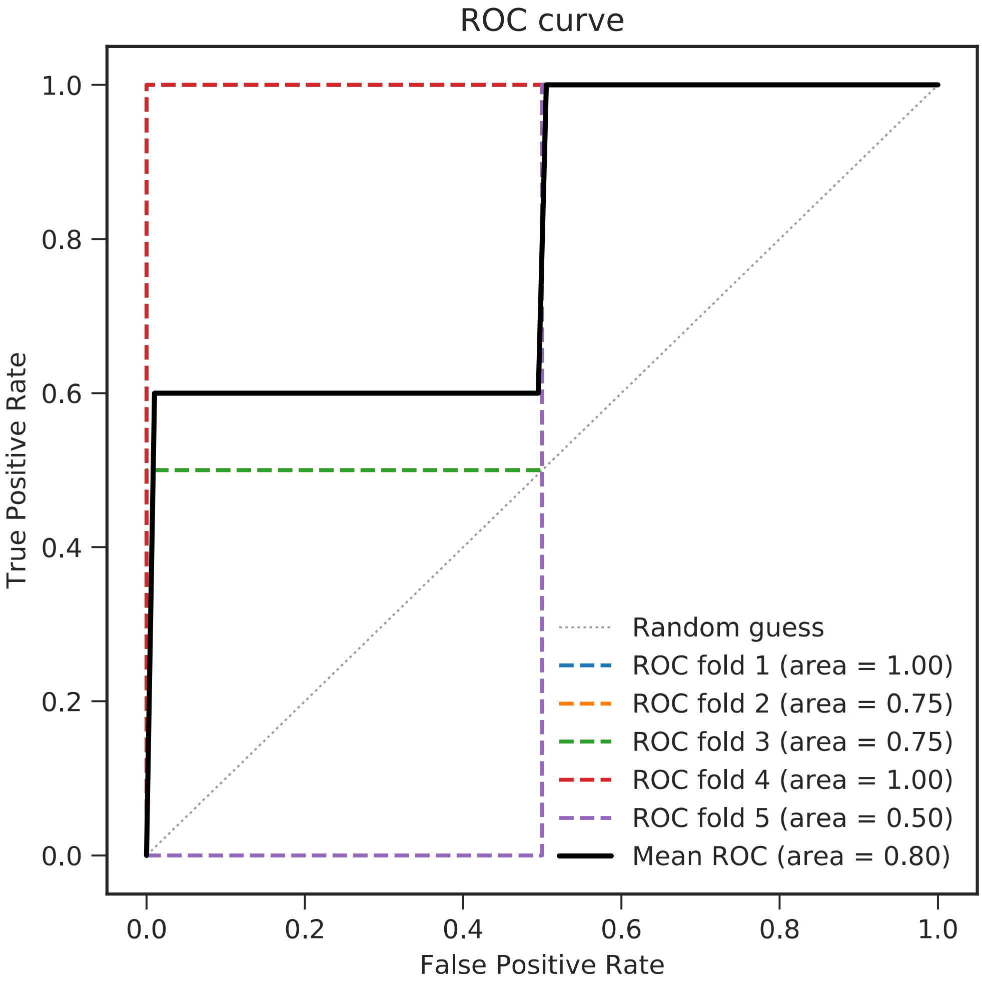

Figure S11: Correlation between dietary intakes and microbiota in the whole dataset. (A) Correlation of dietary intakes with bacterial genus-level abundance. (B) Correlation of dietary intakes with bacterial species-level diversity. (C) Correlation of dietary intakes with fungal genus-level abundance. (D) Correlation of dietary intakes with fungal species-level diversity. *Q* values were generated using the Bonferroni method. + indicates q-values between 0.05 and 0.1; * indicates *q*-values less than 0.05.

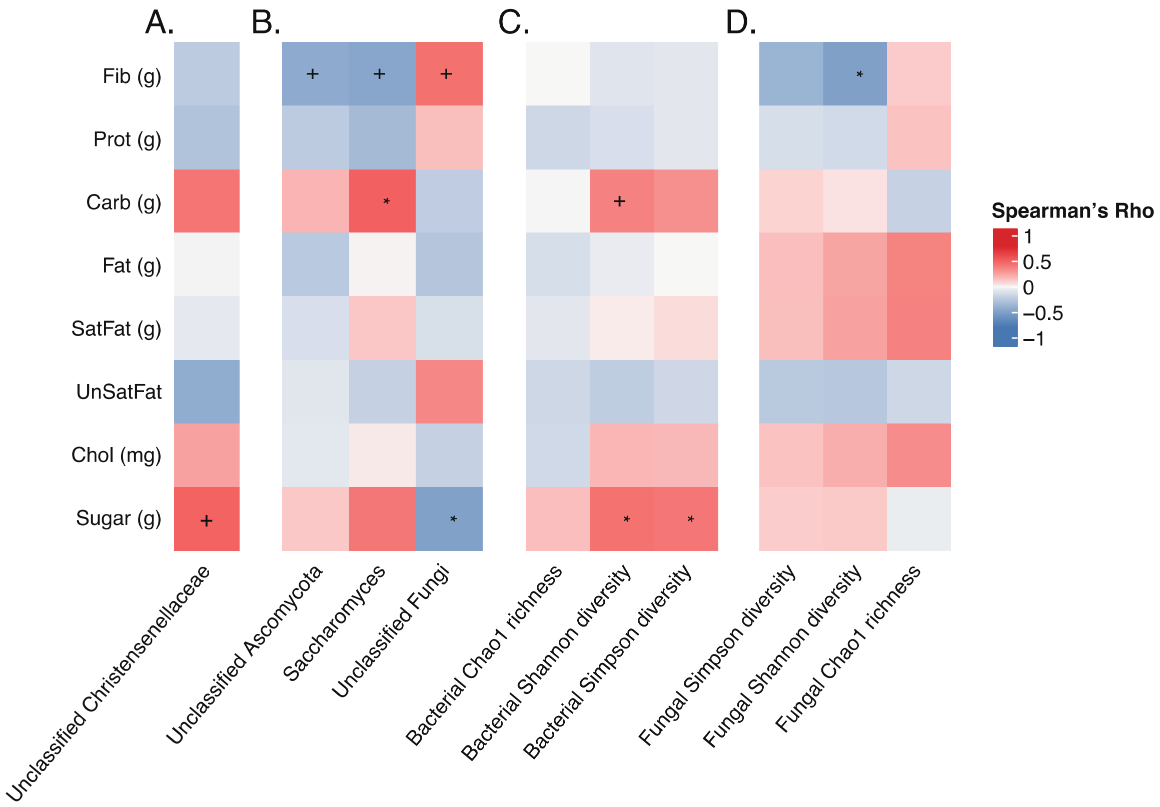

Table S1: Median values of α-diversity indices.

	Index	NW CON	OWOB CON	NW PWS	OWOB PWS		CON	PWS
All bacterial species	Chao1	93.5	86.59	99.1	101.61		92.6	100.72
	Shannon	2.44	2.55	2.17	2.16		2.49	2.17
	Simpson	0.83	0.83	0.82	0.79		0.83	0.82
Actinobacteria	Chao1	4.00^{a}	3.50^{a}	7.67^{b}	6.25^{ab}		4.00^{a}	7.00^{b}
	Shannon	0.3	0.47	0.77	0.32		0.4	0.52
	Simpson	0.14	0.26	0.45	0.14		0.19	0.28
Bacteroidetes	Chao1	18	13	16	19.25		17	16.17
	Shannon	1.28	1.16	0.92	0.75		1.22	0.9
	Simpson	0.6	0.56	0.42	0.35		0.58	0.42
Firmicutes	Chao1	59.5	55.71	62.5	60.45		58.17	61.9
	Shannon	2.09	2.25	2.2	2.15		2.14	2.2
	Simpson	0.8	0.81	0.82	0.79		0.8	0.81
Lentisphaerae	Chao1	0	0	0	0		0	0
	Shannon	0	0	0	0		0	0
	Simpson	1	1	1	1		1	1
Proteobacteria	Chao1	7	7.75	7	7.75		7	7
	Shannon	0.93^{a}	0.89^{ab}	0.78^{ab}	0.45^{b}		0.93^{a}	0.59^{b}
	Simpson	0.53^{a}	0.51^{a}	0.42^{ab}	0.18^{b}		0.53^{a}	0.30^{b}
Tenericutes	Chao1	1	0	1	1		1	1
	Shannon	0	0	0	0		0	0
	Simpson	0.03^{a}	1.00^{b}	0.19^{ab}	0.00^{a}		0.49	0
Verrucomicrobia	Chao1	1.00^{ab}	1.00^{a}	1.00^{ab}	1.00^{b}		1	1
	Shannon	0	0	0	0		0	0
	Simpson	0	0	0	0		0	0
All fungal species	Chao1	8	7	8.25	9		7	9
	Shannon	0.84	0.54	0.73	0.34		0.68	0.47
	Simpson	0.39	0.29	0.38	0.17		0.38	0.21
Ascomycota	Chao1	5	5	5	5		5	5
	Shannon	0.71	0.15	0.67	0.79		0.48	0.76
	Simpson	0.45	0.05	0.42	0.48		0.24	0.48
Basidiomycota	Chao1	1.00^{ab}	1.00^{a}	1.00^{ab}	3.00^{b}		1	2
	Shannon	0.00^{ab}	0.00^{a}	0.00^{ab}	0.30^{b}		0	0.03
	Simpson	0	0.33	0.07	0.39		0	0.17

Comparison between the CON and PWS group: Wilcoxon test (two-sided); comparisons between subgroups: Dunn test (Bonferroni-adjusted p values). a, b, c, d In the same row, values for the four subgroups and for the two groups with different letter superscripts mean significant difference ($p<0.05$)

Table S2: Median abundance and occurrence frequency of differentially abundant bacterial and fungal taxa.

Model	Predictor	Median abundance						Occurrence frequency					
		NW CON	OWOB CON	NW PWS	OWOB PWS	CON	PWS	NW CON	OWOB CON	NW PWS	OWOB PWS	CON	PWS
Differentially abundant bacterial genera: PWS vs. CON	Akkermansia	7.88E-03	1.11E-02	3.11E-04	5.31E-04	7.88E-03	3.11E-04	0.94	0.63	0.87	1.00	0.85	0.92
	Anaerostipes	2.05E-04	3.35E-04	4.83E-05	2.66E-04	2.52E-04	7.62E-05	1.00^{a}	0.75^{ab}	0.60^{b}	1.00^{ab}	0.92	0.76
	Clostridium (Ruminococcaceae)	0.00E+00^{a}	1.60E-04^{ab}	1.04E-04^{ab}	2.44E-04^{b}	5.10E-05	1.51E-04	0.47	0.75	0.67	1.00	0.58	0.80
	Dorea	1.01E-03	1.56E-03	5.18E-04	8.50E-04	1.01E-03	6.07E-04	1.00	1.00	1.00	1.00	1.00	1.00
	Haemophilus	1.08E-03	4.71E-04	2.89E-04	3.22E-04	8.46E-04	3.08E-04	0.88	0.88	0.93	1.00	0.88	0.96
	Oscillospira	2.95E-02	2.11E-02	9.05E-03	1.22E-02	2.33E-02^{a}	1.12E-02^{b}	1.00	1.00	1.00	1.00	1.00	1.00
	Prevotella	2.22E-04^{ab}	1.65E-04^{a}	4.86E-04^{b}	2.78E-04^{ab}	2.11E-04^{a}	4.14E-04^{b}	0.94	0.75	1.00	1.00	0.88	1.00
	Propionibacterium	0.00E+00^{a}	0.00E+00^{ab}	1.74E-05^{c}	2.46E-05^{bc}	0.00E+00^{a}	1.74E-05^{b}	0.00^{a}	0.00^{ab}	0.73^{c}	0.60^{bc}	0.00^{a}	0.68^{b}
	SMB53	4.46E-04^{ab}	3.88E-03^{a}	6.26E-04^{b}	3.00E-04^{ab}	9.02E-04	5.29E-04	1.00	1.00	0.87	1.00	1.00	0.92
	Staphylococcus	0.00E+00^{a}	0.00E+00^{ac}	4.14E-05^{b}	5.31E-05^{bd}	0.00E+00^{a}	4.85E-05^{b}	0.06^{a}	0.00^{ac}	0.73^{b}	0.80^{bd}	0.04^{a}	0.76^{b}
	Unclassified Christensenellaceae	2.16E-04	3.07E-04	7.74E-05	1.44E-04	2.16E-04	8.63E-05	1.00	0.88	0.80	0.80	0.96	0.80
	Unclassified Enterobacteriaceae	0.00E+00^{a}	1.08E-04^{ab}	5.65E-04^{b}	3.06E-04^{b}	2.87E-05^{a}	3.93E-04^{b}	0.47^{a}	0.88^{ab}	1.00^{b}	1.00^{ab}	0.62^{a}	1.00^{b}
	Unclassified RF39	1.66E-04	0.00E+00	1.55E-04	1.96E-05	0.00E+00	8.93E-05	0.59	0.13	0.67	0.70	0.46	0.68
Differentially abundant bacterial genera: OWOB CON vs. NW CON	Alistipes	2.66E-02	1.02E-02	1.46E-02	1.75E-02	2.07E-02	1.55E-02	1.00	1.00	0.93	1.00	1.00	0.96
	Bacteria_unclassified genera	4.57E-03	5.00E-04	1.40E-03	1.70E-03	1.40E-03	1.40E-03	0.94	0.88	1.00	0.90	0.92	0.96
	Bifidobacterium	4.08E-03^{ab}	2.31E-02^{a}	1.74E-03^{b}	9.40E-03^{ab}	6.02E-03	4.94E-03	1.00	1.00	1.00	1.00	1.00	1.00
	Bilophila	1.24E-03	9.16E-05	7.92E-04	4.65E-04	8.58E-04	5.53E-04	0.94	0.50	0.60	0.70	0.81	0.64
	Blautia	8.47E-03	1.73E-02	1.13E-02	8.18E-03	1.07E-02	8.33E-03	1.00	1.00	1.00	1.00	1.00	1.00
	Collinsella	1.10E-04	8.97E-04	2.90E-04	1.64E-04	1.17E-04	2.18E-04	0.76	0.63	0.80	0.90	0.73	0.84
	Dialister	0.00E+00^{a}	3.38E-03^{b}	1.71E-05^{a}	9.55E-04^{ab}	1.12E-04	1.45E-04	0.47	1.00	0.53	0.90	0.62	0.68
	Dorea	1.01E-03	1.56E-03	5.18E-04	8.50E-04	1.01E-03	6.07E-04	1.00	1.00	1.00	1.00	1.00	1.00
	Holdemania	8.61E-05	1.31E-04	8.07E-05	1.23E-04	9.02E-05	9.71E-05	0.94	0.75	0.87	1.00	0.88	0.92
	SMB53	4.46E-04^{ab}	3.88E-03^{a}	6.26E-04^{ab}	3.00E-04^{b}	9.02E-04	5.29E-04	1.00	1.00	0.87	1.00	1.00	0.92
	Sutterella	1.78E-02^{ab}	1.06E-02^{ab}	3.91E-03^{a}	2.48E-02^{b}	1.65E-02	1.11E-02	1.00	1.00	0.93	1.00	1.00	0.96
	Turicibacter	8.61E-05^{ab}	3.21E-04^{a}	9.14E-05^{ab}	1.42E-05^{b}	9.02E-05	6.90E-05	0.82	1.00	0.80	0.60	0.88	0.72
Differentially abundant bacterial genera: OWOB PWS vs. NW PWS	Anaerostipes	2.05E-04	3.35E-04	4.83E-05	2.66E-04	2.52E-04	7.62E-05	1.00^{a}	0.75^{ab}	0.60^{b}	1.00^{ab}	0.92	0.76
	Bacteroides	3.39E-01	3.57E-01	2.69E-01	3.60E-01	3.39E-01	3.08E-01	1.00	1.00	1.00	1.00	1.00	1.00
	Bifidobacterium	4.08E-03^{ab}	2.31E-02^{a}	1.74E-03^{b}	9.40E-03^{ab}	6.02E-03	4.94E-03	1.00	1.00	1.00	1.00	1.00	1.00
	Clostridium (Clostridiaceae)	1.21E-03	1.31E-03	7.07E-04	5.28E-04	1.21E-03	7.07E-04	1.00	1.00	1.00	1.00	1.00	1.00
	Clostridium (Lachnospiraceae)	4.83E-03	6.38E-03	4.49E-03	3.21E-03	4.98E-03	4.14E-03	1.00	1.00	1.00	1.00	1.00	1.00
	Clostridium (Ruminococcaceae)	0.00E+00^{a}	1.60E-04^{ab}	1.04E-04^{ab}	2.44E-04^{b}	5.10E-05	1.51E-04	0.47	0.75	0.67	1.00	0.58	0.80
	Coprococcus	5.93E-03	1.00E-02	4.87E-03	9.31E-03	9.14E-03	6.96E-03	1.00	1.00	1.00	1.00	1.00	1.00
	Dialister	0.00E+00^{a}	3.38E-03^{b}	1.71E-05^{a}	9.55E-04^{ab}	1.12E-04	1.45E-04	0.47	1.00	0.53	0.90	0.62	0.68
	Escherichia	8.05E-05	2.26E-04	3.52E-04	5.75E-05	1.96E-04	1.47E-04	0.76	0.75	1.00	0.90	0.77	0.96
	Parabacteroides	7.93E-03	8.65E-03	3.63E-03	5.60E-03	7.93E-03	3.96E-03	0.88	1.00	0.87	0.80	0.92	0.84
	Prevotella	2.22E-04^{ab}	1.65E-04^{a}	4.86E-04^{b}	2.78E-04^{ab}	2.11E-04^{a}	4.14E-04^{b}	0.94	0.75	1.00	1.00	0.88	1.00
	Sutterella	1.78E-02^{ab}	1.06E-02^{ab}	3.91E-03^{a}	2.48E-02^{b}	1.65E-02	1.11E-02	1.00	1.00	0.93	1.00	1.00	0.96
	Turicibacter	8.61E-05^{ab}	3.21E-04^{a}	9.14E-05^{ab}	1.42E-05^{b}	9.02E-05	6.90E-05	0.82	1.00	0.80	0.60	0.88	0.72
Differentially abundant bacterial genera: PWS vs. CON	Candida	7.43E-03	0.00E+00	8.70E-02	6.05E-02	2.08E-03^{a}	6.71E-02^{b}	0.73	0.43	0.90	0.89	0.61	0.89
	Mrakia	0.00E+00	0.00E+00	0.00E+00	0.00E+00	0.00E+00^{a}	0.00E+00^{b}	0.00	0.00	0.40	0.22	0.00^{a}	0.32^{b}
	Saccharomyces	9.07E-02^{ab}	8.32E-01^{a}	1.40E-04^{b}	8.70E-04^{b}	2.23E-01^{a}	2.81E-04^{b}	1.00	1.00	0.50	0.67	1.00^{a}	0.58^{b}
	Unclassified Agaricomycetes	0.00E+00	0.00E+00	0.00E+00	0.00E+00	0.00E+00^{a}	0.00E+00^{b}	0.00	0.00	0.20	0.44	0.00^{a}	0.32^{b}
	Unclassified Ascomycota	5.11E-03	2.17E-03	6.45E-03	2.72E-04	3.64E-03	7.32E-04	1.00	0.86	0.90	0.89	0.94	0.89
	Unclassified Basidiomycota	0.00E+00^{a}	0.00E+00^{a}	2.34E-05^{ab}	7.73E-04^{b}	0.00E+00^{a}	2.64E-04^{b}	0.09^{a}	0.14^{ab}	0.50^{ab}	0.78^{b}	0.11^{a}	0.63^{b}
Differentially abundant bacterial genera: OWOB CON vs. NW CON	Candida	7.43E-03	0.00E+00	8.70E-02	6.05E-02	2.08E-03^{a}	6.71E-02^{b}	0.73	0.43	0.90	0.89	0.61	0.89
	Cyberlindnera	0.00E+00	0.00E+00	0.00E+00	0.00E+00	0.00E+00	0.00E+00	0.27	0.43	0.40	0.33	0.33	0.37
	Fungi_unclassified genera	3.98E-01	9.44E-02	2.90E-01	7.89E-01	2.50E-01	7.47E-01	1.00	1.00	1.00	1.00	1.00	1.00
	Saccharomyces	9.07E-02^{ab}	8.32E-01^{a}	1.40E-04^{b}	8.70E-04^{b}	2.23E-01^{a}	2.81E-04^{b}	1.00	1.00	0.50	0.67	1.00^{a}	0.58^{b}
	Unclassified Ascomycota	5.11E-03	2.17E-03	6.45E-03	2.72E-04	3.64E-03	7.32E-04	1.00	0.86	0.90	0.89	0.94	0.89
Differentially abundant fungal genera: OWOB PWS vs. NW PWS	Fungi_unclassified genera	3.98E-01	9.44E-02	2.90E-01	7.89E-01	2.50E-01	7.47E-01	1.00	1.00	1.00	1.00	1.00	1.00
	Saccharomyces	9.07E-02^{ab}	8.32E-01^{a}	1.40E-04^{b}	8.70E-04^{b}	2.23E-01^{a}	2.81E-04^{b}	1.00	1.00	0.50	0.67	1.00^{a}	0.58^{b}
	Unclassified Ascomycota	5.11E-03	2.17E-03	6.45E-03	2.72E-04	3.64E-03	7.32E-04	1.00	0.86	0.90	0.89	0.94	0.89
	Unclassified Basidiomycota	0.00E+00^{a}	0.00E+00^{a}	2.34E-05^{ab}	7.73E-04^{b}	0.00E+00^{a}	2.64E-04^{b}	0.09^{a}	0.14^{ab}	0.50^{ab}	0.78^{b}	0.11^{a}	0.63^{b}

Comparison of abundance between the CON and PWS group: Wilcoxon test (two-sided); comparisons of abundance between the subgroups: Dunn test (adjusted p values). Comparison of occurrence frequency between CON and PWS: Fisher's Exact test; comparisons of occurrence between the subgroups: Fisher's Exact test with Bonferroni correction. a, b, c, d in the same row, values for the four subgroups and for the two groups with different letter superscripts mean significant difference ($p<0.05$).

Table S3: Median values of nutrition intakes.

	NW CON	OWOB CON	NW PWS	OWOB PWS	CON	PWS
Fib (g)	20.2	14.2	18.1	19.7	16.5	19.0
Prot (g)	60.7	68.2	69.6	71.9	64.4	70.6
Carb (g)	227.9^{a}	222.8^{ab}	175.4^{b}	195.1^{ab}	225.3^{a}	189.2^{b}
Fat (g)	202.2	204.5	201.3	206.2	202.2	203.6
SatFat (g)	201.6	196.2	194.8	197.8	197.8	195.1
UnSatFat	373.2	373.4	376.5	374.0	373.2	376.3
Chol (mg)	147.0	212.7	161.9	187.3	162.8	178.5
Sugar (g)	199.9	207.5	197.6	200.0	203.5	197.9

Comparison between CON and PWS: Wilcoxon test (two-sided); comparisons between the subgroups: Dunn test (adjusted p values). a, b, c, d in the same row, values for the four subgroups and for the two groups with different letter superscripts mean significant difference (p<0.05).

Table S4: Logistic regression for the association between CHO intake, group and dichotomized abundance of differentially abundant bacterial and fungal taxa.

	Estimates in logistic regression model					
Genus-level abundance (Ref: below-the-median)	CHO (Ref: above-the-median)		Group (Ref: PWS)		CHO * Group	
	Odds ratio (95% confidence interval)	p value	Odds ratio (95% conf	p value	Odds ratio (95% cor	p value
Staphylococcus	1.08 (0.15, 7.64)	0.936	0.02 (0.00, 0.27)	**0.003**	0 (0, NA)	0.999
Unclassified Enterobacteriaceae	0.34 (0.03, 3.56)	0.37	0.03 (0.00, 0.35)	**0.005**	8.17 (0.40, 166.29)	0.172
Propionibacterium	1.44 (0.25, 8.46)	0.687	0 (0, NA)	0.998	0.69 (0, NA)	1
Prevotella	1.95 (0.32, 12.01)	0.472	0.25 (0.04, 1.47)	0.125	0.41 (0.03, 5.73)	0.508
Oscillospira	0.42 (0.07, 2.36)	0.323	2.40 (0.42, 13.60)	0.323	1.00 (0.09, 11.63)	1
Dorea	0.33 (0.06, 1.87)	0.209	0.86 (0.15, 4.82)	0.861	2.14 (0.19, 24.23)	0.539
Clostridium (Ruminococcaceae)	0.48 (0.07, 3.09)	0.437	0.30 (0.05, 1.91)	0.2	0.34 (0.02, 6.55)	0.473
SMB53	7.88 (0.79, 78.67)	0.079	10.00 (1.00, 100.46)	0.05	0.15 (0.01, 2.63)	0.193
UnclassifiedRF39	3.06 (0.54, 17.46)	0.209	1.48 (0.27, 8.27)	0.654	0.22 (0.02, 2.56)	0.227
Akkermansia	2.10 (0.32, 13.61)	0.437	7.20 (1.07, 48.64)	**0.043**	0.20 (0.02, 2.54)	0.214
Anaerostipes	0.25 (0.04, 1.47)	0.125	1.10 (0.19, 6.29)	0.915	2.18 (0.19, 25.52)	0.534
Unclassified Christensenellaceae	0.14 (0.02, 0.94)	**0.043**	0.61 (0.09, 4.02)	0.608	2.36 (0.18, 31.29)	0.516
Haemophilus	7.88 (0.79, 78.67)	0.079	12.83 (1.26, 130.51)	**0.031**	0.07 (0.00, 1.22)	0.068
Saccharomyces	0 (0, NA)	0.999	6.00 (0.70, 51.10)	0.101	INF (0, NA)	0.999
Candida	0 (0, NA)	0.999	0 (0, NA)	0.999	INF (0, NA)	0.999
Mrakia	1.25 (0.16, 9.54)	0.83	0 (0, NA)	0.999	0.80 (0, NA)	1
Unclassified Basidiomycota	0.56 (0.08, 4.14)	0.57	0.09 (0.01, 0.84)	**0.035**	0 (0, NA)	0.999
Unclassified Ascomycota	0.25 (0.03, 1.82)	0.171	1.31 (0.19, 9.10)	0.783	3.05 (0.19, 48.86)	0.431
Unclassified Agaricomycetes	4.29 (0.39, 47.63)	0.236	0 (0, NA)	0.999	0.23 (0, NA)	1

INF: infinite value; NA: not available.

Table S5: Logistic regression for the association between hyperphagia scores, group and dichotomized abundance of differentially abundant bacterial and fungal taxa.

Genus-level abundance (Ref: below-the-median)	Estimates in logistic regression model						
		Hyperphagia score (Ref: above-the-median)		Group (Ref: PWS)		Score * Group	
		Odds ratio (95% confidence interval)	p value	Odds ratio (95% conf	p value	Odds ratio (95% conf	p value
Staphylococcus	Behaviour score	0.15 (0.01, 1.50)	0.105	0.02 (0.00, 0.29)	**0.005**	0 (0, NA)	0.999
Staphylococcus	Drive score	0.23 (0.03, 1.63)	0.141	0 (0, NA)	0.999	INF (0, NA)	0.999
Staphylococcus	Severity score	0.27 (0.04, 1.86)	0.183	0.05 (0.00, 0.62)	**0.02**	0 (0, NA)	0.998
Staphylococcus	Total score	0.23 (0.03, 1.63)	0.141	0.02 (0.00, 0.22)	0.017	0 (0, NA)	0.999
Unclassified Enterobacteriaceae	Behaviour score	2.75 (0.40, 18.88)	0.303	0 (0, NA)	0.999	INF (0, NA)	0.999
Unclassified Enterobacteriaceae	Drive score	4.50 (0.44, 46.17)	0.205	0.07 (0.01, 0.75)	0.071	0.65 (0.02, 17.60)	0.648
Unclassified Enterobacteriaceae	Severity score	2.31 (0.22, 24.32)	0.486	0.10 (0.01, 1.08)	0.058	0.58 (0.02, 16.84)	0.75
Unclassified Enterobacteriaceae	Total score	4.50 (0.44, 46.17)	0.205	0.06 (0.01, 0.57)	0.015	1.00 (0.04, 26.91)	1
Propionibacterium	Behaviour score	0.53 (0.10, 2.98)	0.474	0 (0, NA)	0.999	1.88 (0.00, NA)	1
Propionibacterium	Drive score	1.17 (0.21, 6.56)	0.861	0 (0, NA)	0.999	0.86 (0.00, NA)	1
Propionibacterium	Severity score	3.82 (0.38, 38.83)	0.258	0 (0, NA)	0.999	0.26 (0.00, NA)	1
Propionibacterium	Total score	1.17 (0.21, 6.56)	0.861	0 (0, NA)	0.999	0.86 (0.00, NA)	1
Prevotella	Behaviour score	0.32 (0.05, 2.11)	0.236	0 (0, NA)	0.999	INF (0, NA)	0.999
Prevotella	Drive score	0.85 (0.14, 4.99)	0.856	0 (0, NA)	0.999	INF (0, NA)	0.999
Prevotella	Severity score	0.38 (0.06, 2.46)	0.31	0 (0, NA)	0.999	INF (0, NA)	0.999
Prevotella	Total score	0.38 (0.06, 2.24)	0.283	0 (0, NA)	0.999	INF (0, NA)	0.999
Oscillospira	Behaviour score	1.25 (0.24, 6.44)	0.79	12.00 (1.05, 136.79)	0.045	0.17 (0.01, 2.84)	0.215
Oscillospira	Drive score	0.64 (0.12, 3.53)	0.611	0.90 (0.15, 5.26)	0.907	8.43 (0.70, 101.65)	0.093
Oscillospira	Severity score	0.63 (0.10, 4.18)	0.631	2.36 (0.31, 17.85)	0.407	1.97 (0.12, 31.21)	0.631
Oscillospira	Total score	0.64 (0.12, 3.53)	0.611	2.25 (0.44,11.52)	0.33	2.07 (0.19, 22.34)	0.547
Dorea	Behaviour score	0.63 (0.13, 3.07)	0.562	2.50 (0.34, 18.33)	0.367	0.64 (0.05, 7.52)	0.723
Dorea	Drive score	0.38 (0.07, 2.03)	0.256	2.63 (0.40, 17.46)	0.318	0.79 (0.06, 9.77)	0.79
Dorea	Severity score	0.40 (0.06, 2.63)	0.34	1.50 (0.20, 11.24)	0.693	2.04 (0.13, 31.62)	0.611
Dorea	Total score	0.38 (0.07, 2.03)	0.256	1.31 (0.26, 6.64)	0.742	2.03 (0.20, 21.15)	0.553
Clostridium (Ruminococcaceae)	Behaviour score	0.39 (0.07, 2.13)	0.277	0.13 (0.02, 1.09)	0.06	4.09 (0.32, 52.20)	0.278
Clostridium (Ruminococcaceae)	Drive score	0.36 (0.07, 1.97)	0.24	0.36 (0.06, 2.19)	0.27	1.15 (0.10, 12.88)	0.912
Clostridium (Ruminococcaceae)	Severity score	0.67 (0.11, 3.99)	0.657	0.33 (0.04, 2.56)	0.291	1.21 (0.08, 17.88)	0.889
Clostridium (Ruminococcaceae)	Total score	0.36 (0.07, 1.97)	0.24	0.16 (0.03, 0.92)	**0.04**	4.28 (0.39, 46.98)	0.235
SMB53	Behaviour score	3.50 (0.64, 19.20)	0.149	4.00 (0.55, 29.17)	0.171	0.34 (0.03, 3.93)	0.385
SMB53	Drive score	1.00 (0.20, 5.12)	1	1.50 (0.27, 8.45)	0.646	1.83 (0.17, 19.45)	0.615
SMB53	Severity score	1.18 (0.20, 6.93)	0.856	6.29 (0.58, 68.42)	0.131	0.26 (0.01, 4.97)	0.259
SMB53	Total score	2.00 (0.39, 10.31)	0.407	3.00 (0.57, 15.77)	0.194	0.50 (0.05, 5.06)	0.557
Unclassified RF39	Behaviour score	1.60 (0.33, 7.85)	0.562	0.40 (0.06, 2.93)	0.367	1.56 (0.13, 18.36)	0.723
Unclassified RF39	Drive score	1.31 (0.26, 6.64)	0.742	0.53 (0.09, 3.03)	0.525	1.13 (0.11, 11.99)	0.92
Unclassified RF39	Severity score	0.48 (0.08, 2.81)	0.413	0.16 (0.02, 1.73)	0.131	8.38 (0.44, 160.30)	0.158
Unclassified RF39	Total score	1.31 (0.26, 6.64)	0.742	0.38 (0.07, 2.03)	0.256	2.03 (0.20, 21.15)	0.553
Akkermansia	Behaviour score	0.62 (0.12, 3.22)	0.572	1.87 (0.28, 12.31)	0.517	2.41 (0.21, 27.39)	0.478
Akkermansia	Drive score	2.75 (0.51, 14.86)	0.24	1.65 (0.26, 10.31)	0.592	1.97 (0.17, 23.52)	0.592
Akkermansia	Severity score	3.47 (0.56, 21.35)	0.18	3.90 (0.49, 30.76)	0.196	0.36 (0.02, 5.37)	0.457
Akkermansia	Total score	0.64 (0.12, 3.53)	0.611	1.50 (0.30, 7.53)	0.622	4.28 (0.39, 46.98)	0.235
Anaerostipes	Behaviour score	0.44 (0.09, 2.28)	0.33	6.00 (0.54, 66.17)	0.142	0.38 (0.02, 6.35)	0.497
Anaerostipes	Drive score	0.49 (0.09, 2.66)	0.408	3.43 (0.52, 22.80)	0.202	0.77 (0.06, 9.47)	0.835
Anaerostipes	Severity score	1.18 (0.20, 6.93)	0.856	6.29 (0.58, 68.42)	0.131	0.26 (0.01, 4.97)	0.37
Anaerostipes	Total score	0.49 (0.09, 2.66)	0.408	2.67 (0.49, 14.46)	0.256	1.00 (0.09, 10.92)	1
Unclassified Christensenellaceae	Behaviour score	0.63 (0.13, 3.07)	0.562	2.50 (0.34, 18.33)	0.367	0.64 (0.05, 7.52)	0.64
Unclassified Christensenellaceae	Drive score	0.38 (0.07, 2.03)	0.256	0.88 (0.16, 4.87)	0.879	3.81 (0.35, 41.57)	0.273
Unclassified Christensenellaceae	Severity score	0.94 (0.16, 5.46)	0.943	1.88 (0.25, 14.08)	0.541	0.87 (0.06, 12.44)	0.918
Unclassified Christensenellaceae	Total score	0.38 (0.07, 2.03)	0.256	0.88 (0.18, 4.34)	0.87	4.00 (0.39, 41.40)	0.245
Haemophilus	Behaviour score	1.71 (0.34, 8.68)	0.515	2.67 (0.39, 18.17)	0.316	0.69 (0.06, 7.59)	0.76
Haemophilus	Drive score	0.49 (0.09, 2.66)	0.408	3.43 (0.52, 22.80)	0.202	0.77 (0.06, 9.47)	0.835
Haemophilus	Severity score	0.17 (0.02, 1.68)	0.128	0.67 (0.09, 4.99)	0.693	16.71 (0.78, 357.48)	0.072
Haemophilus	Total score	0.49 (0.09, 2.66)	0.408	1.71 (0.34, 8.68)	0.515	2.04 (0.20, 21.42)	0.552
Saccharomyces	Behaviour score	0 (0, NA)	0.999	INF (0, NA)	0.999	1.29 (0, NA)	1
Saccharomyces	Drive score	0.64 (0.05, 8.62)	0.739	4.50 (0.37, 54.16)	0.236	20.22 (0.44, 937.25)	0.124
Saccharomyces	Severity score	0.83 (0.06, 11.28)	0.891	INF (0, NA)	1	0 (0, NA)	1
Saccharomyces	Total score	0 (0, NA)	0.999	10.00 (0.74, 135.33)	0.083	INF (0, NA)	0.999
Candida	Behaviour score	0.09 (0.01, 1.03)	0.053	0.06 (0.00, 0.81)	**0.034**	7.50 (0.29, 194.11)	0.225
Candida	Drive score	1.71 (0.23, 12.89)	0.601	0 (0, NA)	0.999	INF (0, NA)	0.999
Candida	Severity score	4.29 (0.39, 47.63)	0.236	0 (0, NA)	1	INF (0, NA)	1
Candida	Total score	0.89 (0.11, 7.02)	0.911	0 (0, NA)	0.999	INF (0, NA)	0.999
Mrakia	Behaviour score	0.43 (0.06, 3.22)	0.41	0 (0, NA)	0.999	2.33 (0, NA)	1
Mrakia	Drive score	0.58 (0.08, 4.39)	0.601	0 (0, NA)	0.999	1.71 (0, NA)	1
Mrakia	Severity score	2.25 (0.31, 16.41)	0.424	0 (0, NA)	1	0.44 (0, NA)	1
Mrakia	Total score	1.13 (0.14, 8.88)	0.911	0 (0, NA)	0.999	0.89 (0, NA)	1
Unclassified Basidiomycota	Behaviour score	3.50 (0.47, 25.90)	0.22	0.20 (0.02, 2.39)	0.203	0.13 (0.00, 4.65)	0.264
Unclassified Basidiomycota	Drive score	2.50 (0.34, 18.33)	0.367	0.83 (0.08, 8.24)	0.876	0 (0, NA)	0.998
Unclassified Basidiomycota	Severity score	6.00 (0.54, 66.17)	0.143	0 (0, NA)	1	INF (0, NA)	1
Unclassified Basidiomycota	Total score	4.29 (0.39, 47.63)	0.236	0.86 (0.09, 8.08)	0.893	0 (0, NA)	0.998
Unclassified Ascomycota	Behaviour score	0.29 (0.04, 2.11)	0.22	0.50 (0.06, 4.09)	0.518	21.00 (1.12, 393.93)	0.042
Unclassified Ascomycota	Drive score	2.67 (0.39, 18.17)	0.316	2.67 (0.25, 28.44)	0.417	0.68 (0.04, 12.93)	0.794
Unclassified Ascomycota	Severity score	4.00 (0.55, 29.17)	0.171	0 (0, NA)	1	INF (0, NA)	1
Unclassified Ascomycota	Total score	0.80 (0.11, 6.10)	0.83	1.60 (0.17, 15.27)	0.683	2.25 (0.11, 46.46)	0.6
Unclassified Agaricomycetes	Behaviour score	1.17 (0.17, 8.09)	0.876	0 (0, NA)	0.999	0.86 (0.00, NA)	1
Unclassified Agaricomycetes	Drive score	0.58 (0.08, 4.39)	0.601	0 (0, NA)	0.999	1.71 (0, NA)	1
Unclassified Agaricomycetes	Severity score	0.23 (0.02, 2.59)	0.236	0 (0, NA)	1	4.29 (0, NA)	1
Unclassified Agaricomycetes	Total score	1.13 (0.14, 8.88)	0.911	0 (0, NA)	0.999	0.89 (0, NA)	1

INF: infinite value; NA: not available.
